# Supplementary material for: QTLs and Candidate Loci Associated with Drought Tolerance Traits of Kaybonnet x ZHE733 Recombinant Inbred Lines Rice Population
Source: Int J Mol Sci. 2023 Oct 14;24(20):15167. doi: 10.3390/ijms242015167 (PMC10606886; doi:10.3390/ijms242015167)
Supplement: Supplementary file 1 [file ijms-24-15167-s001.zip › Supplementary Figure S1_Manuscript_Rice_Identification of QTLs and Candidate Genes.pdf]

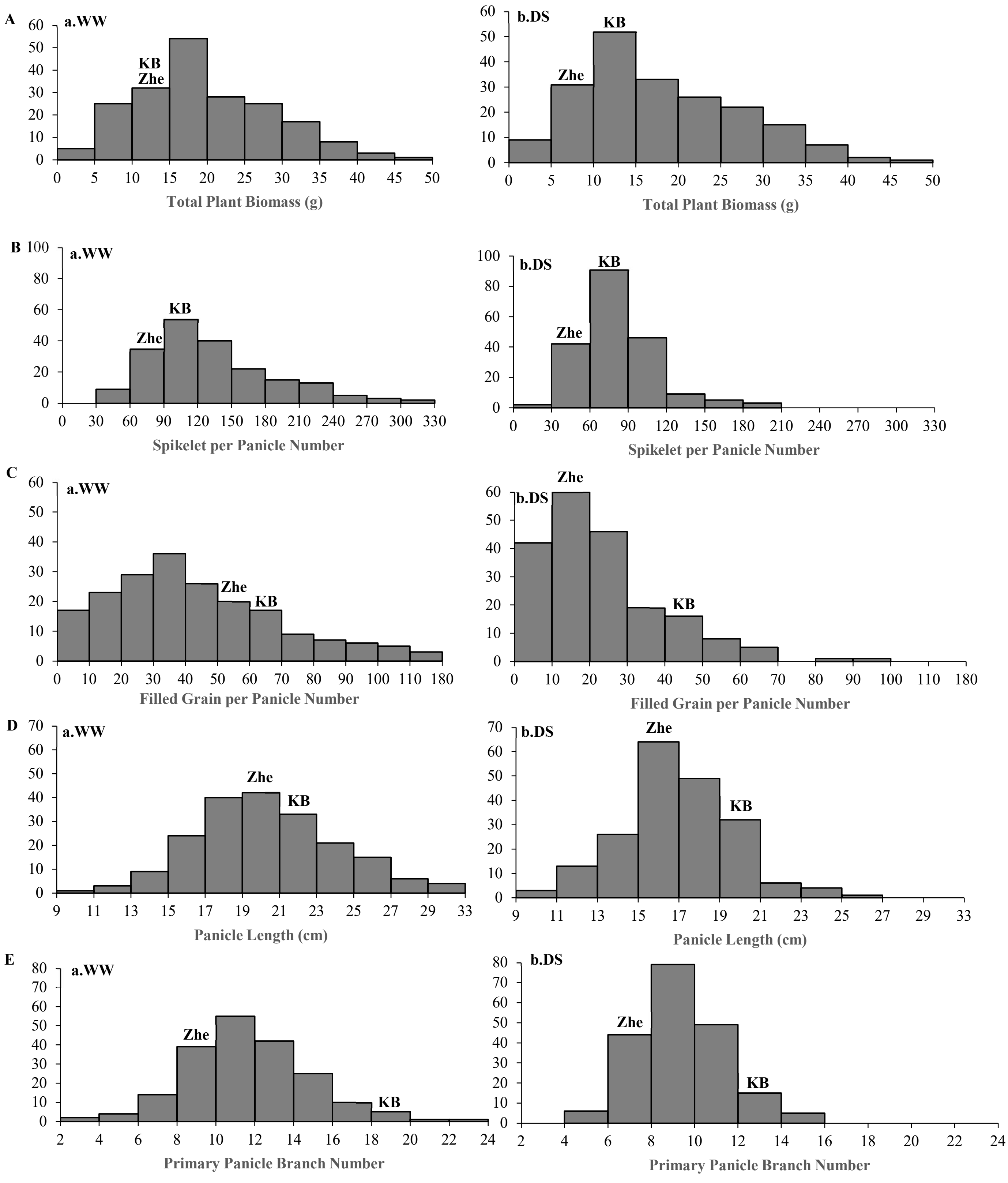

Supplementary Figure 1. Frequency distribution of total plant biomass (A), spikelet per panicle number (B), filled grain per panicle number (C), panicle length (D), and primary panicle branch number (E) under WW (a) and DS (b)

conditions
