# Supplementary material for: QTLs and Candidate Loci Associated with Drought Tolerance Traits of Kaybonnet x ZHE733 Recombinant Inbred Lines Rice Population
Source: Int J Mol Sci. 2023 Oct 14;24(20):15167. doi: 10.3390/ijms242015167 (PMC10606886; doi:10.3390/ijms242015167)
Supplement: Supplementary file 1 [file ijms-24-15167-s001.zip › Supplementary Figure S2_Manuscript_Rice_Identification of QTLs and Candidate Genes.pdf]

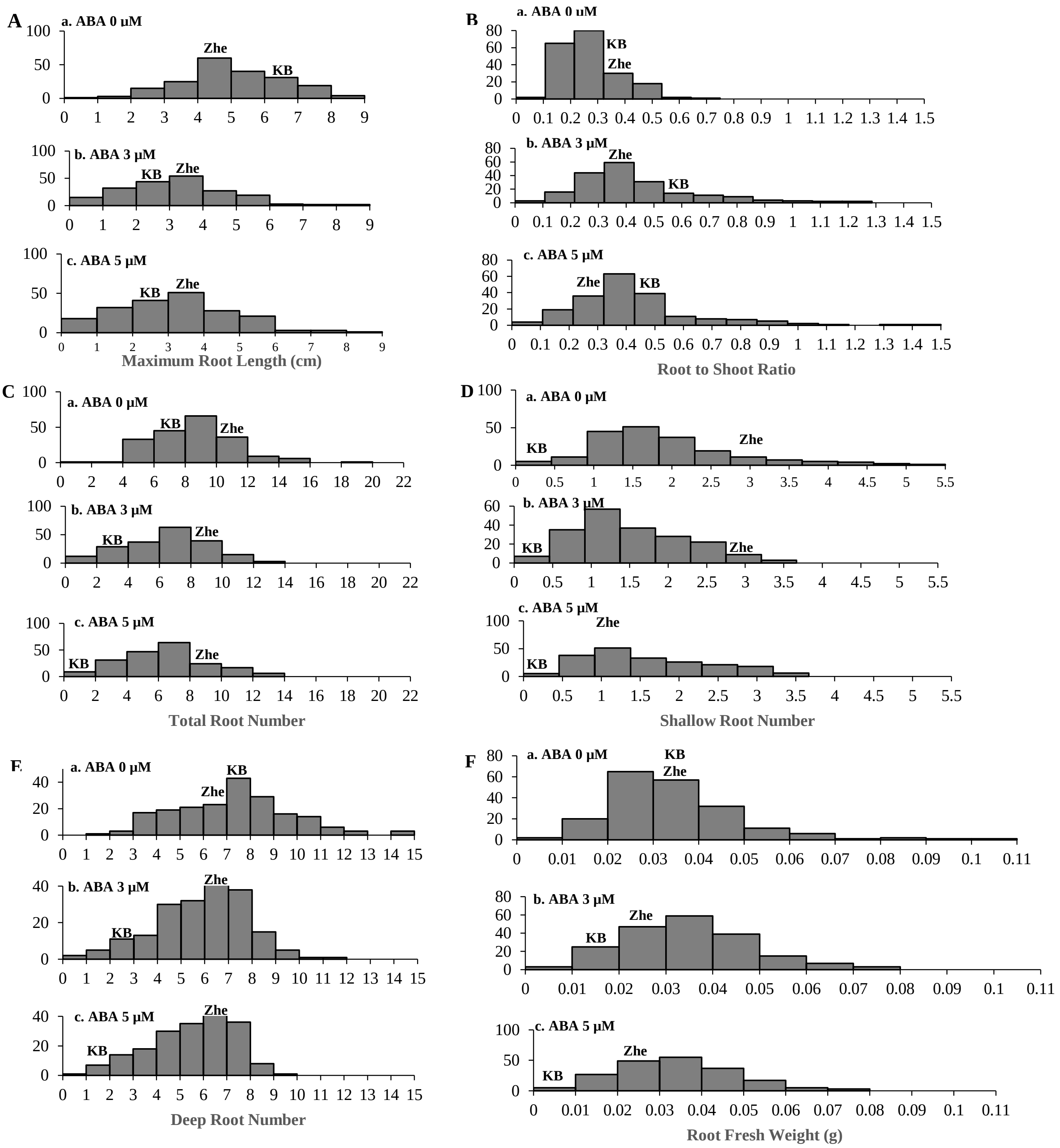

Supplementary Figure 2. Frequency distribution of root length (A), root to shoot ratio (B), total root number (C), shallow root number (D), deep root number (E), and root fresh weight (F) under control (ABA 0 µM) (a), ABA 3 µM (b), and ABA 5 µM (c)
