# Supplementary material for: QTLs and Candidate Loci Associated with Drought Tolerance Traits of Kaybonnet x ZHE733 Recombinant Inbred Lines Rice Population
Source: Int J Mol Sci. 2023 Oct 14;24(20):15167. doi: 10.3390/ijms242015167 (PMC10606886; doi:10.3390/ijms242015167)
Supplement: Supplementary file 1 [file ijms-24-15167-s001.zip › Supplementary Table S1_Manuscript_Rice_Identification of QTLs and Candidate Genes_4-18-2022.pdf]

Table S1. The average and range values of morphological traits of K/Z RIL population

Traits	Treatments	Kaybonnet	ZHE733	K/Z RIL Population	
				Average	Range
Plant height (cm)	WW	98 ± 0.3	51 ± 0.1	60.03 ± 0.3	28 - 98
	DS	84 ± 0.2	31 ± 0.1	29.99 ± 0.4	10 - 73
Tiller number	WW	5 ± 0.2	8 ± 0.3	8.11 ± 0.1	3 - 22
	DS	4 ± 0.1	7 ± 0.3	6.98 ± 0.2	2 - 19
Heading day (day)	-	91 ± 0.3	71 ± 0.1	87 ± 0.1	65 - 109
Flag leaf width (cm)	DS	1.7 ± 0.02	1.3 ± 0.02	1.2 ± 0.3	0.7 - 2
Leaf rolling score	DS	2 ± 0.1	4 ± 0.2	2.8 ± 0.2	1 - 4
