# Supplementary material for: QTLs and Candidate Loci Associated with Drought Tolerance Traits of Kaybonnet x ZHE733 Recombinant Inbred Lines Rice Population
Source: Int J Mol Sci. 2023 Oct 14;24(20):15167. doi: 10.3390/ijms242015167 (PMC10606886; doi:10.3390/ijms242015167)
Supplement: Supplementary file 1 [file ijms-24-15167-s001.zip › Supplementary Table S2_Manuscript_Rice_Identification of QTLs and Candidate Genes_4-18-2022.pdf]

Table S2. The average and range values of root architectural traits of K/Z RIL population under ABA conditions

Traits	Treatments	Kaybonnet	ZHE733	K/Z RIL Population	
				Average	Range
Root length (cm)	ABA 0 µM	6.570 ± 0.2	4.611 ± 0.1	4.428 ± 0.2	0.500 - 8.933
	ABA 3 µM	2.500 ± 0.2	3.800 ± 0.2	3.600 ± 0.3	0.094 - 8.651
	ABA 5 µM	2.050 ± 0.1	3.678 ± 0.1	3.491 ± 0.3	0.100 - 8.367
Root to shoot ratio	ABA 0 µM	0.363 ± 0.07	0.324 ± 0.05	0.259 ± 0.08	0.079 - 0.669
	ABA 3 µM	0.587 ± 0.03	0.303 ± 0.08	0.336 ± 0.09	0.013 - 1.216
	ABA 5 µM	0.432 ± 0.06	0.298 ± 0.05	0.335 ± 0.03	0.014 - 1.411
Total root number	ABA 0 µM	6.600 ± 0.2	10.222 ± 0.5	8.362 ± 0.4	1.667 - 18.667
	ABA 3 µM	2.500 ± 0.1	9.667 ± 0.3	6.986 ± 0.2	1.000 - 11.333
	ABA 5 µM	1.750 ± 0.2	9.200 ± 0.7	6.600 ± 0.5	1.000 - 12.333
Shallow root number	ABA 0 µM	0.200 ± 0.05	3.333 ± 0.1	1.649 ± 0.1	0.000 - 5.667
	ABA 3 µM	0.000 ± 0.00	3.177 ± 0.2	1.092 ± 0.1	0.000 - 3.540
	ABA 5 µM	0.000 ± 0.00	3.005 ± 0.2	1.047 ± 0.3	0.000 - 3.667
Deep root number	ABA 0 µM	7.400 ± 0.4	6.889 ± 0.3	7.043 ± 0.5	1.667 - 14.667
	ABA 3 µM	2.500 ± 0.2	6.490 ± 0.4	6.391 ± 0.3	0.000 - 11.000
	ABA 5 µM	1.750 ± 0.1	6.195 ± 0.2	6.155 ± 0.2	0.000 - 9.333
Root fresh weight	ABA 0 µM	0.032 ± 0.01	0.031± 0.02	0.034 ± 0.02	0.001 - 0.114
	ABA 3 µM	0.016 ± 0.01	0.029 ± 0.01	0.033 ± 0.01	0.000 - 0.083
	ABA 5 µM	0.005 ± 0.002	0.027 ± 0.001	0.026 ± 0.001	0.000 - 0.078
