# Supplementary material for: QTLs and Candidate Loci Associated with Drought Tolerance Traits of Kaybonnet x ZHE733 Recombinant Inbred Lines Rice Population
Source: Int J Mol Sci. 2023 Oct 14;24(20):15167. doi: 10.3390/ijms242015167 (PMC10606886; doi:10.3390/ijms242015167)
Supplement: Supplementary file 1 [file ijms-24-15167-s001.zip › Supplementary Table S3_Manuscript_Rice_Identification of QTLs and Candidate Genes_4-18-2022.pdf]

Table S3. QTLs identified for morphological and yield traits under DS and WW conditions and root architectural traits under ABA conditions with IciMapping

Trait name	QTLs	Chromo-some	LOD	PVE (%)	Peak Marker	Position (cM)	Candidate gene position (bp)	Number of genes within 25 kb of the marker closest to the QTL peak	Gene annotation
PHC	*qPHC1.1*	1	10.57	4.85	KZ14152	43	38480787	9	Protein phosphorylation
PHC	*qPHC2.1*	2	24.42	13.81	KZ23918	546	27023516	7	Transport.Major_Intrinsic_Proteins.PIP
PHC	*qPHC2.2*	2	17.19	8.63	KZ24204	552	29443430	11	Protein phosphorylation
RL_ABA3	*qRLA3-2.3*	2	28.85	0.61	KZ24639	236	33179587	5	Stress.abiotic.heat
RL_ABA5	*qRLA5-2.2*	2	20.72	0.59					
TPBD	*qTPBD3.1*	3	10.57	6.48	KZ35574	19	32079708	6	Signaling.receptor_kinases.leucine_rich_repeat_XI
RL_ABA3	*qRLA3-3.1*	3	32.94	0.61					
RL_ABA5	*qRLA5-3.1*	3	24.79	0.59					
PPBC	*qPPBC4.1*	4	4.73	10.34	KZ44474	137	436771	7	PS.photorespiration.hydroxypyruvate_reductase
TPBC	*qTPBC4.2*	4	3.58	2.78	KZ45735	226	26174168	7	PS.lightreaction.photosystem_II.PSII_polypeptide_subunits
RL_ABA3	*qRLA3-4.2*	4	22.34	0.61					
RL_ABA5	*qRLA5-4.2*	4	14.21	0.59					
PLD	*qPLD4.2*	4	2.70	6.08	KZ44511	286	1233282	7	ERF domain containing protein. (Os04t0649100-02);APETALA2 transcription factor, Seed shattering through abscission zone (AZ) development (Os04t0649100-03)
FGC	*qFGC5.1*	5	5.74	1.12	KZ57234	481	28101481	12	Drought-responsive ethylene response factor 10, drought-responsive ERF 10, ethylene response factor 84, APETALA2/ethylene-responsive element binding protein 97
FGD	*qFGD5.1*	5	2.53	4.74					
RL_ABA3	*qRLA3-5.1*	5	25.08	0.61					
RL_ABA5	*qRLA5-5.1*	5	16.95	0.59					

Table S3. QTLs identified for morphological and yield traits under DS and WW conditions and root architectural traits under ABA conditions with IciMapping (Continued)

Trait name	QTLs	Chromo-some	LOD	PVE (%)	Peak Marker	Position (cM)	Candidate gene position (bp)	Number of genes within 25 kb of the marker closest to the QTL peak	Gene annotation
FGC	*qFGC6.2*	6	3.71	0.87	KZ68491	230	29755505	9	Control of rice architecture via BR signaling (Os06t0704300-01)_Tetrapyrrole biosynthetic process
TPBC	*qTPBC6.2*	6	3.01	2.66					
RL_ABA3	*qRLA3-6.1*	6	26.24	0.61					
RL_ABA5	*qRLA5-6.1*	6	18.12	0.59					
FGC	*qFGC7.1*	7	3.06	1.27	KZ79333	283	28219138	10	Lipid_metabolism.lipid_degradation.lipases
SPC	*qSPC7.1*	7	2.58	9.88					
SPC	*qSPC7.2*	7	4.62	6.37	KZ79821	91	9822937	7	Protein of unknown function DUF594 domain containing protein. (Os07t0269400-00)
SPD	*qSPD7.1*	7	4.85	2.12	KZ77844	265	912849	9	Stress biotic_Endoplasmic reticulum protein, Regulation of sugar partitioning in carbon-demanding young leaves and developing leaf sheaths (Os07t0116300-01)
PLC	*qPLC7.1*	7	5.05	9.62	KZ78019	107	11608339	13	Regulation of nutrient metabolism and endosperm development (Os07t0296900-01)
PPBC	*qPPBC7.1*	7	2.91	6.27					
SPC	*qSPC8.1*	8	3.40	5.75	KZ90034	115	26147631	11	Embryo and endosperm development (Os08t0525500-01)_carbohydrate metabolic process_UDP-glucuronic acid 4-epimerase 2
PPBC	*qPPBC8.1*	8	2.66	7.25					
FGD	*qFGD8.1*	8	2.78	3.79	KZ89547	357	3507836	6	RNA.processing.RNA_helicase_DEAD-box RNA helicase, Pre-mRNA splicing under cold stress (Os08t0159900-01)
RL_ABA3	*qRLA3-8.1*	8	24.48	0.61					
RL_ABA5	*qRLA5-8.1*	8	16.35	0.59					

Table S3. QTLs identified for morphological and yield traits under DS and WW conditions and root architectural traits under ABA conditions with IciMapping (Continued)

Trait name	QTLs	Chromo-some	LOD	PVE (%)	Peak Marker	Position (cM)	Candidate gene position (bp)	Number of genes within 25 kb of the marker closest to the QTL peak	Gene annotation
TPBD	*qTPBD9.1*	9	3.37	2.24	KZ101571	341	4220164	7	Carbohydrate metabolic process (GO:0005975)
RL_ABA5	*qRLA5-9.1*	9	13.57	0.59					
SPD	*qSPD10.1*	10	3.03	0.98	KZ102164	300	12048509	6	Intracellular protein transport (GO:0006886)
RL_ABA3	*qRLA3-10.1*	10	20.11	0.61					
TPBC	*qTPBC10.1*	10	7.25	5.09	KZ102869	112	3205210	10	RNA.transcription
TPBD	*qTPBD10.2*	10	6.71	4.31	KZ102866	118	3156402	5	MYB transcription factor
RL_ABA3	*qRLA3-10.3*	10	23.86	0.61					
TPBD	*qTPBD10.3*	10	21.82	8.49	KZ102900	302	3719254	10	Similar to Chalcone and stilbene synthases, N-terminal domain containing protein. (Os10t0158400-00)
TPBD	*qTPBD12.2*	12	3.19	6.34	KZ105668	412	953389	10	Late embryogenesis abundant protein, LEA-14 (IPR004864)
