# Supplementary material for: QTLs and Candidate Loci Associated with Drought Tolerance Traits of Kaybonnet x ZHE733 Recombinant Inbred Lines Rice Population
Source: Int J Mol Sci. 2023 Oct 14;24(20):15167. doi: 10.3390/ijms242015167 (PMC10606886; doi:10.3390/ijms242015167)
Supplement: Supplementary file 1 [file ijms-24-15167-s001.zip › Supplementary Table S4_Manuscript_Rice_Identification of QTLs and Candidate Genes.pdf]

Table S4. Candidate drought resistance genes within QTL regions for generating gene expression with RT-qPCR

No.	Genes	Forward	Reverse
**Internal control gene**			
1	Ubiquitin	5'-CGCAAGTACAACCAGGACAA-3'	5'-GCTGTGACCACACTTCTTCTT-3'
**Candidate drought resistance genes within QTL regions with known annotations to be responsive to drought stress**			
1	LOC_Os01g66270	5'-GGATGTACGGCGCTCAC-3'	5'-ACATCAGGATCTCCATGTCATC-3'
2	LOC_Os02g54160	5'-GCTCCTATTGAGGACCCTATCA-3'	5'-GGTATCATTCTCCCAGCCAAAG-3'
3	LOC_Os03g56280	5'-TGCTGGCGTTGTGGAAT-3'	5'-GCACGACCTAACCTCACTTT-3'
4	LOC_Os04g44190	5'-GAGGAGGACTTGGTGATTGATG-3'	5'-GCCGAGGTCAATGTACAAGAA-3'
5	LOC_Os05g49010	5'-CCGCGACGAACTGTGAT-3'	5'-GATCACTCCTGCGTCACTTT-3'
6	LOC_Os05g49210	5'-CTTCAAGACGGCGGAGAAG-3'	5'-CCGACGAGCAATCCCATATT-3'
7	LOC_Os05g49240	5'-GCCACTACGAAGACCTCAC-3'	5'-TCCAGTATCCCTGCGTCT-3'
8	LOC_Os06g49080	5'-GTCAACCATTTGGGAGCTTTC-3'	5'-GGAGGAGTTACCATCCCATTAAG-3'
9	LOC_Os07g02520	5'-CACAAGGTACTGGTGGTTCTC-3'	5'-CGTTGCTGCTGTAGATGTAGT-3'
10	LOC_Os08g06344	5'-CTGTTCCAGAGGACCTGAAAG-3'	5'-CGCTTCCACCATATCCTGTT-3'
11	LOC_Os09g08120	5'-GGGAGCGACGACGTTAAG-3'	5'-GTCGGCTCCAACTCCAAC-3'
12	LOC_Os10g41460	5'-CTACCGCGATAGGTGGGA-3'	5'-ATCTTGTGGCGGCACTG-3'
13	LOC_Os11g30760	5'-CCGTCACGTTCCGCTAC-3'	5'-TGGACGATCGATGATGGTTG-3'
14	LOC_Os12g02700	5'-CACCCTTCAGAGCCCATAA-3'	5'-GATGGCAAGGACGAGGAG-3'
15	LOC_Os12g29330	5'-GCTTGTGGTGCACTACCTC-3'	5'-TGAAGAAGTACCTCTCCTGCTC-3'
**Candidate drought resistance genes from the traits with high LOD and PVE with unknown annotations**			
1	LOC_Os02g44590	5'-GACGAGCACCTAATGAAGGAG-3'	5'-GCCGGCATCGTGTAGATAG-3'
2	LOC_Os02g44599	5'-CAACCTCGGCGATCTTCTG-3'	5'-GGACCTTCGCGTACATGG-3'
3	LOC_Os02g44610	5'-CATGAGAGAGACACTGCCATAG-3'	5'-AAAGAGAAGTCGGCGATCTG-3'
4	LOC_Os02g44620	5'-TGATCTGTTCTTCGGTGGAAAG-3'	5'-CCTAAGCAGGGCATGATTGT-3'
5	LOC_Os02g44630	5'-GGTCTTGGTGCTGAGATTGT-3'	5'-GTCCCTGGCATTCCTCTTG-3'
6	LOC_Os02g44642	5'-GACCTGAAACTACCGAACCTG-3'	5'-GTCCCTGAAGTCGCAAATCT-3'
7	LOC_Os10g07030	5'-ACAAGCACGCAAGCAGT-3'	5'-CCTCGTCAAGTCCTCCCT-3'
8	LOC_Os10g07040	5'-CAACGATCCTCGCCATCG-3'	5'-CGGTAAGGTGCTCGCTTT-3'
9	LOC_Os10g07050	5'-CAGCTGCCCATGCATCC-3'	5'-CGTCCTCTCTGTCGCATTT-3'
10	LOC_Os10g07060	5'-GGTCCCTTAGTCCATCAGATCA-3'	5'-TACTGCCTGTTCCGTTCCT-3'
11	LOC_Os10g07080	5'-CCCGCACCTAGCATCTTG-3'	5'-GCGGGATGTCGACGTATG-3'
