# Supplementary material for: QTLs and Candidate Loci Associated with Drought Tolerance Traits of Kaybonnet x ZHE733 Recombinant Inbred Lines Rice Population
Source: Int J Mol Sci. 2023 Oct 14;24(20):15167. doi: 10.3390/ijms242015167 (PMC10606886; doi:10.3390/ijms242015167)
Supplement: Supplementary file 1 [file ijms-24-15167-s001.zip › Supplementary Table S5_Manuscript_Rice_Identification of QTLs and Candidate Genes.pdf]

Table S5. Phenotypic correlation between grain yield components and morphological traits of the K/Z RIL population under WW & DS conditions

	**HD**	**FLW**	**DLR**	**PH-WW**	**PH-DS**	**TN-WW**	**TN-DS**	**SP-WW**	**SP-DS**	**PL-WW**	**PL-DS**	**PPB-WW**	**PPB-DS**	**FG-WW**	**FG-DS**	**TPB-WW**
**FLW**	-0.132															
**DLR**	0.078	0.067														
**PH-WW**	-0.119	-0.044	-0.124													
**PH-DS**	-0.154	0.282	0.078	0.315												
**TN-WW**	0.100	0.063	-0.091	-0.080	-0.168											
**TN-DS**	0.071	0.021	-0.096	0.039	-0.170	0.466										
**SP-WW**	0.083	-0.072	0.064	0.063	-0.117	0.098	0.003									
**SP-DS**	0.129	0.040	-0.025	0.003	-0.027	0.178	0.037	0.537								
**PL-WW**	-0.026	-0.003	0.050	0.385	0.099	-0.008	-0.043	0.457	0.250							
**PL-DS**	-0.061	0.214	0.000	0.088	0.277	-0.043	-0.112	0.045	0.399	0.121						
**PPB-WW**	0.084	-0.084	0.111	0.149	0.025	0.038	-0.036	0.535	0.252	0.523	0.004					
**PPB-DS**	0.063	0.240	-0.079	-0.010	0.050	0.052	0.021	0.166	0.513	0.056	0.621	0.063				
**FG-WW**	0.088	0.020	0.165	0.055	0.124	0.066	-0.090	0.340	0.489	0.176	-0.028	0.196	-0.038			
**FG-DS**	0.029	0.064	0.114	0.041	0.125	0.085	-0.078	0.284	0.471	0.153	0.026	0.132	-0.043	0.888		
**TPB-WW**	-0.121	0.096	-0.061	-0.080	-0.057	0.086	0.080	-0.070	-0.107	-0.093	-0.020	-0.053	0.070	-0.269	-0.286	
**TPB-DS**	-0.054	-0.056	-0.099	-0.062	-0.097	0.077	0.019	-0.079	-0.074	-0.108	-0.073	0.027	-0.031	-0.169	-0.203	0.632

*HD, Heading day under WW and DS; FLW, Flag leaf width (cm); DLR, Drought leaf rolling under DS; PH-WW, Plant height (cm) under WW; PH-DS, Plant height (cm) under DS; TN-WW, Productive tiller number under WW; TN-DS, Productive tiller number under DS; SP-WW, Spikelet per panicle number under WW; SP-DS, Spikelet per panicle number under DS; PL-WW, Panicle length (cm) under WW; PL-DS, Panicle length (cm) under DS; PPB-WW, Primary panicle branch number under WW; PPB-DS, Primary panicle branch number under DS; FG-WW, Filled grain per panicle number under WW; FG-DS, Filled grain per panicle number under DS; TPB-WW, Total plant biomass (g) under WW; TPB-DS, Total plant biomass (g) under DS.
