# Supplementary material for: QTLs and Candidate Loci Associated with Drought Tolerance Traits of Kaybonnet x ZHE733 Recombinant Inbred Lines Rice Population
Source: Int J Mol Sci. 2023 Oct 14;24(20):15167. doi: 10.3390/ijms242015167 (PMC10606886; doi:10.3390/ijms242015167)
Supplement: Supplementary file 1 [file ijms-24-15167-s001.zip › Supplementary Table S6_Manuscript_Rice_Identification of QTLs and Candidate Genes.pdf]

Table S6. Phenotypic correlation between grain yield components under WW and DS conditions, and root architectural traits under ABA conditions of the K/Z RIL population

	FG-WW	FG-DS	RL-A0	RL-A3	RL-A5	RSR-A0	RSR-A3	RSR-A5	TRN-A0	TRN-A3	TRN-A5	SRN-A0	SRN-A3	SRN-A5	DRN-A0	DRN-A3	DRN-A5	RFW-A0	RFW-A3
**FG-DS**	0.376																		
**RL-A0**	-0.087	0.086																	
**RL-A3**	-0.062	0.236	0.644																
**RL-A5**	-0.075	0.220	0.619	0.931															
**RSR-A0**	-0.030	0.032	0.722	0.503	0.492														
**RSR-A3**	-0.032	0.128	0.566	0.665	0.640	0.447													
**RSR-A5**	-0.051	0.198	0.526	0.624	0.715	0.376	0.764												
**TRN-A0**	-0.079	0.078	0.305	0.217	0.237	0.087	0.428	0.371											
**TRN-A3**	-0.046	0.246	0.495	0.578	0.572	0.412	0.334	0.390	0.380										
**TRN-A5**	-0.029	0.253	0.418	0.525	0.531	0.369	0.388	0.309	0.430	0.785									
**SRN-A0**	-0.020	-0.092	-0.360	-0.288	-0.249	-0.296	-0.185	-0.152	0.123	-0.188	-0.057								
**SRN-A3**	-0.082	0.017	0.080	-0.075	-0.096	0.188	-0.145	-0.156	0.035	0.326	0.265	0.111							
**SRN-A5**	-0.058	0.069	0.119	-0.058	-0.061	0.028	0.020	-0.067	0.155	0.158	0.382	0.174	0.431						
**DRN-A0**	-0.063	0.135	0.462	0.348	0.350	0.221	0.504	0.439	0.901	0.461	0.460	-0.286	-0.024	0.093					
**DRN-A3**	-0.021	0.234	0.476	0.635	0.642	0.338	0.414	0.477	0.387	0.889	0.695	-0.246	-0.138	-0.048	0.498				
**DRN-A5**	-0.004	0.235	0.398	0.587	0.598	0.385	0.409	0.358	0.398	0.778	0.921	-0.130	0.109	-0.006	0.454	0.767			
**RFW-A0**	-0.029	0.033	0.501	0.360	0.341	0.150	0.284	0.228	0.387	0.253	0.227	-0.150	-0.011	0.042	0.438	0.272	0.225		
**RFW-A3**	0.066	0.267	0.276	0.484	0.448	0.137	0.143	0.192	0.128	0.579	0.463	-0.169	0.059	-0.039	0.215	0.567	0.500	0.289	
**RFW-A5**	0.052	0.212	0.298	0.459	0.478	0.210	0.144	0.176	0.134	0.568	0.598	-0.137	0.111	0.078	0.205	0.536	0.606	0.286	0.858

* FG-WW, Filled grain per panicle number under WW; FG-DS, Filled grain per panicle number under DS; RL-A0, maximum root length under ABA 0 µM; RL-A3, maximum root length under ABA 3 µM; RL-A5, maximum root length under ABA 5 µM; RSR-A0, root to shoot ratio under ABA 0 µM; RSR-A3, root to shoot ratio under ABA 3 µM; RSR-A5, root to shoot ratio under ABA 5 µM; TRN-A0, total root number under ABA 0 µM; TRN-A3, total root number under ABA 3 µM, TRN-A5, total root number under ABA 5 µM; SRN-A0, number of roots with a shallow angle (0-45°) under ABA 0 µM; SRN-A3, number of roots with a shallow angle (0-45°) under ABA 3 µM; SRN-A5, number of roots with a shallow angle (0-45°) under ABA 5 µM; DRN-A0, number of roots with a deep angle (45-90°) under ABA 0 µM; DRN-A3, number of roots with a deep angle (45-90°) under ABA 3 µM; DRN-A5, number of roots with a deep angle (45-90°) under ABA 5 µM; RFW-A0, root fresh weight under ABA 0 µM; RFW-A3, root fresh weight under ABA 3 µM; RFW-A5, root fresh weight under ABA 5 µM.
