# Supplementary figures and images for: QTLs and Candidate Loci Associated with Drought Tolerance Traits of Kaybonnet x ZHE733 Recombinant Inbred Lines Rice Population
Source: Int J Mol Sci. 2023 Oct 14;24(20):15167. doi: 10.3390/ijms242015167 (PMC10606886; doi:10.3390/ijms242015167)

## Slide 1
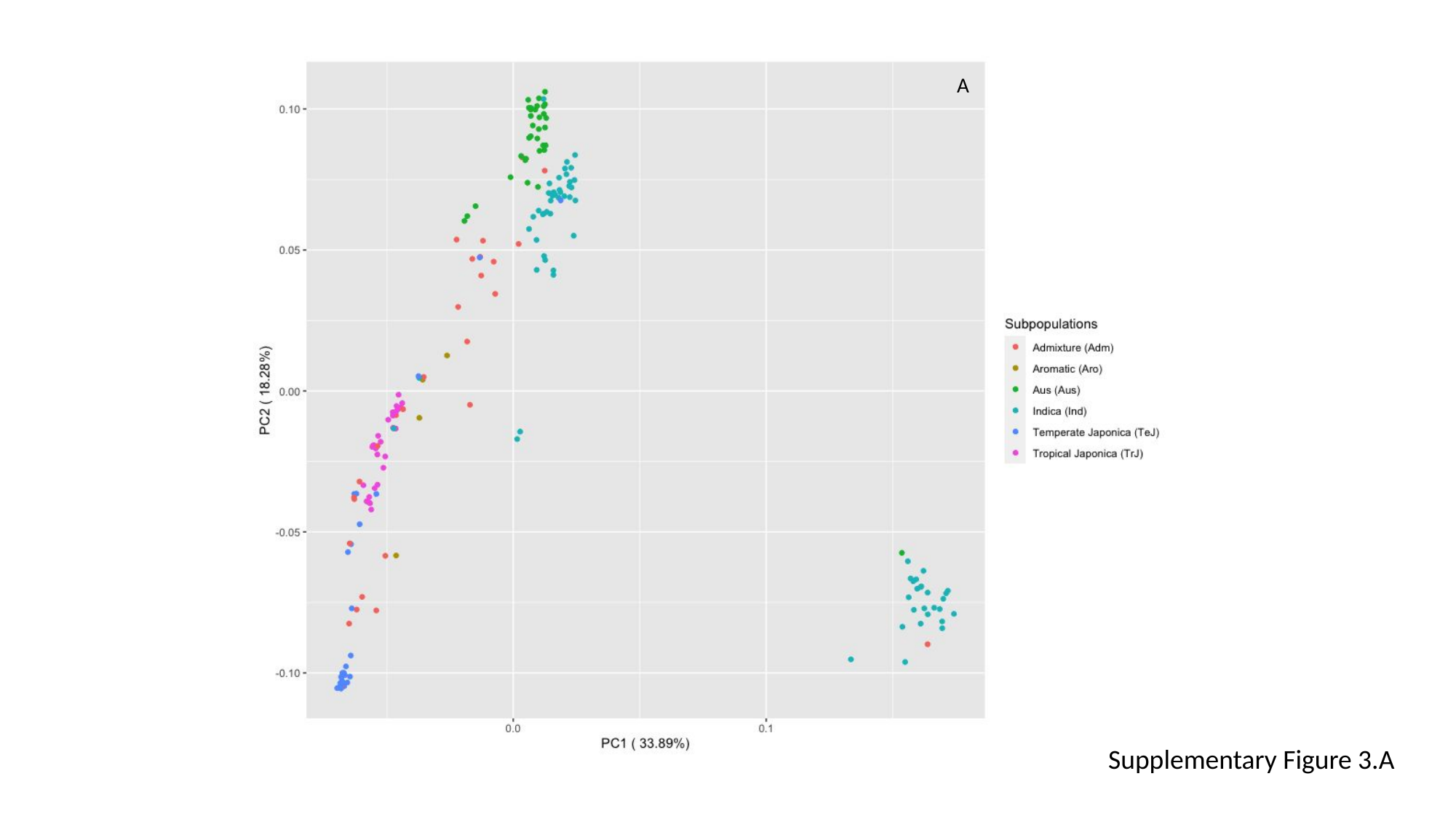

A
Supplementary Figure 3.A

## Slide 2
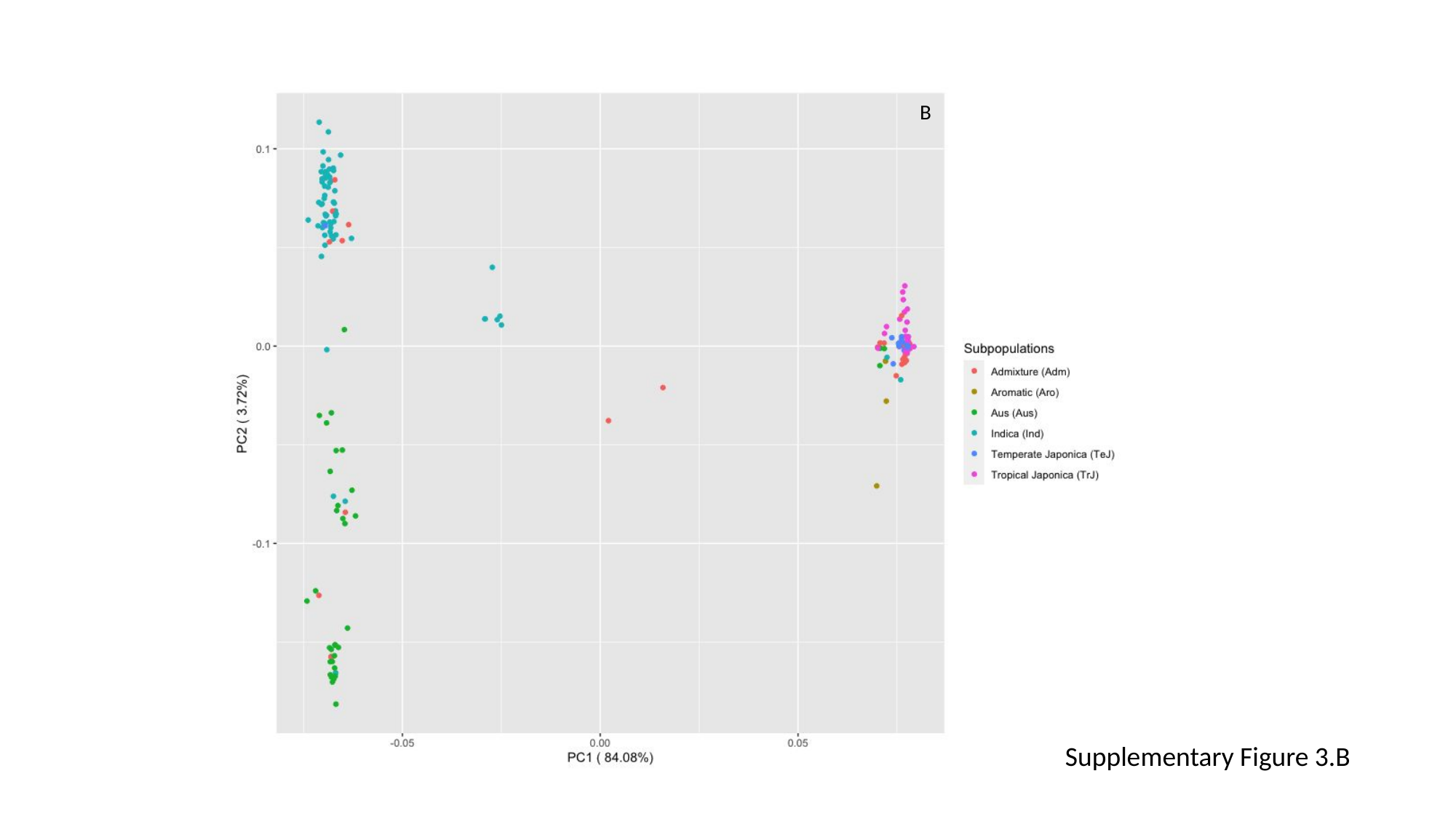

B
Supplementary Figure 3.B

## Slide 3
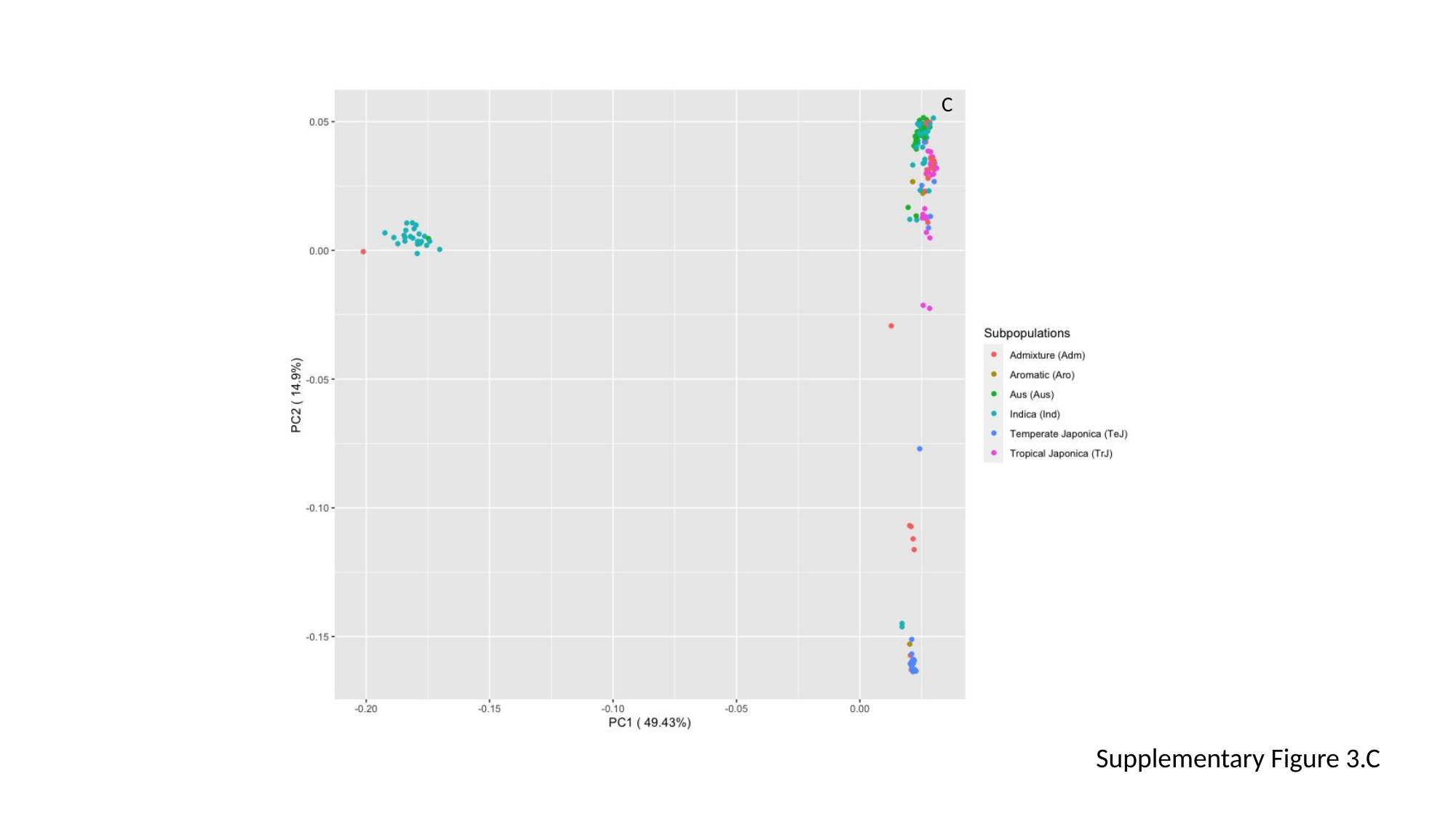

C
Supplementary Figure 3.C

Supplement: Supplementary file 1 [file ijms-24-15167-s001.zip › Supplementary Figure S3_Manuscript_Rice_Identification of QTLs and Candidate Genes.docx.xlsx.pptx]
